# Evolution of surface area and membrane shear modulus of matured human red blood cells during mechanical fatigue

**DOI:** 10.1038/s41598-023-34605-x

**Published:** 2023-05-26

**Authors:** Qiaodong Wei, Xiaolong Wang, Ce Zhang, Ming Dao, Xiaobo Gong

**Affiliations:** 1 Key Laboratory of Hydrodynamics (Ministry of Education), Department of Engineering Mechanics, School of Naval Architecture Ocean and Civil Engineering, Shanghai Jiao Tong University, Shanghai, 200240 China; 2 Institute of Photonics and Photon Technology, State Key Laboratory of Photon-Technology in Western China Energy, Northwest University, Xi’an, 710100 China; 3 Department of Materials Science and Engineering, Massachusetts Institute of Technology, Cambridge, MA 02139 USA; 4 State Key Laboratory of Ocean Engineering, School of Naval Architecture, Ocean and Civil Engineering, Shanghai Jiao Tong University, Shanghai, 200240 China

**Keywords:** Biomedical engineering, Biological physics, Fluid dynamics, Characterization and analytical techniques

## Abstract

Mechanical properties of red blood cells (RBCs) change during their senescence which supports numerous physiological or pathological processes in circulatory systems by providing crucial cellular mechanical environments of hemodynamics. However, quantitative studies on the aging and variations of RBC properties are largely lacking. Herein, we investigate morphological changes, softening or stiffening of single RBCs during aging using an in vitro mechanical fatigue model. Using a microfluidic system with microtubes, RBCs are repeatedly subjected to stretch and relaxation as they squeeze into and out of a sudden contraction region. Geometric parameters and mechanical properties of healthy human RBCs are characterized systematically upon each mechanical loading cycle. Our experimental results identify three typical shape transformations of RBCs during mechanical fatigue, which are all strongly associated with the loss of surface area. We constructed mathematical models for the evolution of surface area and membrane shear modulus of single RBCs during mechanical fatigue, and quantitatively developed an ensemble parameter to evaluate the aging status of RBCs. This study provides not only a novel in vitro fatigue model for investigating the mechanical behavior of RBCs, but also an index closely related to the age and inherent physical properties for a quantitative differentiation of individual RBCs.

## Introduction

In mammals, red blood cells (RBCs) are among the most important cells for sustaining living conditions while continuously traveling through various sizes of circulatory vessels and narrow gaps. During the typical lifespan of 120 days of a human RBC, it changes geometric and mechanical properties with cell aging^1–3^, and exhibits biophysical phenotypes for the diagnosis of various diseases^4,5^. Different from the senescence of nucleated cells, RBCs do not have a nucleus, and thus exhibit unique regulation for cell aging. As RBCs repeatedly squeeze through the microvasculature and submicronic splenic interendothelial slits (IES), and traverse the macro-vasculature, they undergo significant mechanical cycling through large elastic stretching and relaxation^6^. Among one of the most important questions for RBC biology, the effect of mechanical fatigue on the senescence of RBCs has not been addressed quantitatively.

During cell aging, RBCs partially shed their membranes, leading to a change in their morphology from a cup-shaped to a stable discoid biconcave-shaped^7^. RBCs maintain their optimal cellular shapes during long-term circulations by generating microvesicles^8,9^ and regulating cell volume^10^ to remove cellular damage, including membrane damage due to mechanical fatigue and oxidative stress^3^. The analysis of physical characteristics comparing young to aged RBCs^1,2,11^ employs isotope, biotin, or glycated hemoglobin (HbA1c) labeling as markers for cell ages^11,12^ and supports that the volume and surface area decrease monotonically with cell aging. But the observations on the change of membrane shear modulus during cell aging are not consistent that Sutera et al.^13^ observed a significant increase in the elastic modulus of RBCs membrane during cell aging in vivo, while Li et al.^14^ stated that reticulocytes are stiffer than the matured erythrocytes.

Due to the lack of a nucleus and mRNA, RBCs respond mainly to their mechanical microenvironment passively, therefore, the subjection of RBCs to mechanical stimulations during their lifespan is arguably important. It has been noticed that mechanical loading in the spleen plays a crucial role in RBC biology. The spleen-specific IES structure not only facilitates maturation through the removal of reticulocyte organelles^15^, but also contributes to the alteration of the membrane shear modulus, shape transition^5,7,15^ and elimination of aged or diseased RBCs^15–17^. Here, splenic flows with RBCs squeezing through an IES of typically 0.65 μm length and 2–3 μm height are crucial features of their vesiculation, as the density of skeleton-to-membrane connections is vulnerable to reduction under mechanical cycling with extremely narrow slits^18^. This is supported by the discovery that more hemoglobin loss appears in vesicles of RBC vesiculation from individuals with splenic disorders compared to those from healthy individuals in vivo^19^. Furthermore, in blood disorders such as hereditary spherocytosis, the rate of surface area loss in RBCs increases under splenic cyclic loading due to the weakening of the cohesion between the lipid bilayer and cytoskeleton^17^. Therefore, the mechanical cycling through microcapillary vessels and narrow lumens as a standard routine of mechanical stimulations in RBCs’ lifespan implicates an underlying mechanical principle for their maturation and aging. Hence, further investigation is needed to understand the quantitative evolution of the surface area and membrane shear modulus of single RBCs with mechanical cycling, in relation to the process of RBC aging.

Recent studies on the changes of mechanical properties of RBCs adopted mechanical cycling as typical loads to accelerate cell aging in vitro, and various such models were tested. A simple model used rectangular micro channels with a cross-section of $3\times 4$ μm^2^, where the reduction of the deformability of single RBCs was roughly estimated using the Taylor parameter^20^. IES mimicking channels fabricated with microfluidic technologies were set up for circulating RBCs, and the variations in RBC profiles and their deformability were assessed^21^. Amplitude-modulated electro deformation was also used for repeated mechanical stimulation of RBCs to study their mechanical fatigue under hypoxia and ATP depletion^22–24^. To characterize the geometric features of cells (such as their size, surface area, and volume) precisely and separately from their mechanical properties (such as membrane shear modulus) during the mechanical cycling process, more suitable models must be established.

To measure the variations of geometric features and membrane stiffnesses of single RBCs during their mechanical cycling, we propose a microfluidics method to simulate mechanical cycling for single RBCs. A microtube with an inner diameter round 3 μm was connected to a hydraulic system with reversible pressure control. RBCs are squeezed into and out of sudden contractions from a reservoir through the microtube, where RBCs experienced similar restriction fatigue conditions as in a splenic slit. Morphological characteristics (cup-shaped and biconcave-shaped RBCs mainly), geometric parameters (surface area, volume), and mechanical properties (membrane shear modulus) of healthy human RBCs, are measured precisely and recorded instantly with each mechanical cycling of the cell. With the present method, we observed the area loss during cycling and distinctly identified three typical shape transitions of RBCs under fatigue. We have developed mathematical models to study the evolution of surface area and shear modulus of single RBCs during mechanical cycling. These models provide insights into the mechanisms underlying the aging process of RBCs, characterized by surface area loss and stiffness variations. Furthermore, we have defined an ensemble parameter as a novel biophysical marker to assess the health state of individual RBCs at the cellular level. This parameter has the potential to be utilized in clinical practice for the diagnosis of RBC-related diseases.

## Methods

### Mechanical cycling model for single RBCs in vitro

**Figure 1:**
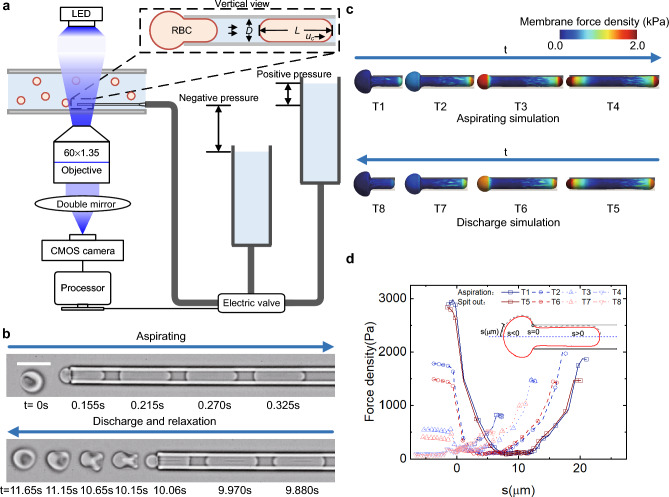
In vitro mechanical cycling model of single RBCs using micro tubular channels. (**a**) Experimental setup. A tubular channel immersed in a large chamber is connected to two different hydrostatic pressure sources through an electric valve. Images of the cells are acquired and recorded with a CMOS camera mounted on the microscope. The experimental system is controlled by a computer program. The region of interest for the dynamical measurement is the steadily moving region of the deformed cell. (**b**) Sequential images of an RBC squeezing into and being pushed out of a microtube under sudden contraction and expansion. The upper part depicts the aspirating process and the lower part is RBC discharge and relaxation. The scale bar is 10 μm. (**c**) Simulated RBC squeezing into and out of the microchannel with the inner diameter of 3 μm. Colors denote the contours of membrane force density on the deformed membrane during mechanical cycling. (**d**) Distributions of membrane force density along deformed membrane during mechanical cycling.

We developed an in vitro single RBC mechanical cycling model (Fig. 1a). In contrast to previous models using microfluidics, which consist of a converge-diverge channel with a smooth-transition region in cross Sections^20^, or employ cyclic tensile loading on an adherent RBC with electric fields of amplitude-modulated electrode^22^, a tubular channel with around 3.0–3.2 µm in diameter is immersed in a large chamber with a suspension of RBCs. The driving pressures of the flows in the channel are shiftable between two different hydrostatic pressures that are higher or lower than the pressure inside the chamber by connecting the tubular channel with two liquid columns at different heights through a three-way electric valve (Fig. 1a). By controlling the pressure in the channel with a compiled program, single RBCs experience a sudden contraction followed by expansion and relaxation (Fig. 1b) repeatably while they are aspirated into or pushed out from the micro channel with fluid flows under hydraulic pressures. With this in vitro mechanical cycling model for single RBCs, we could not only mimic the most severe mechanical fatigue process of RBCs in vivo, but also measure the variations in the surface area, volume, and membrane shear modulus of each RBC during each round of cycling.

The diameter of the tubular channel is optimized to fall within the range of 3.0–3.2 µm to mimic the characteristic size of the smallest capillarities in microcirculation or the IES in spleens and measure the surface area, cell volume, and membrane shear modulus of the single RBC precisely during each cycle of the mechanical fatigue. The Reynolds number of RBCs in the microtube was approximately 0.02. Using the images of each single cell steadily deformed and moving in microtubes under negative pressure during aspiration of the cycling, and assuming that the deformed RBCs under low-Reynolds-number flow in the steadily moving region were axisymmetric, the surface area and volume of the RBC were integrated from the discretized segmentations of the deformed cell profile, i.e. surface area (*A*) and volume (*V*) were taken as the sum of surface areas and volumes of elementary cones, respectively. We performed one-to-one comparison experiments on many RBCs by measuring their surface areas, volumes and membrane shear moduli using both the proposed the microfluidic method and the micropipette aspiration method. Firstly, one cell was aspirated into a microtube until the deformed profiles of the cell in the steady-state region were recorded. Secondly, by changing the pressure difference, the RBC was ejected from the microtube into the chamber. Thirdly, the same RBC was aspirated using a 1.8 μm pipette, and the surface area, cell volume and membrane shear elastic modulus of the RBC were measured. We found that using tubular channels with a diameter in the range of 3.0–3.2 µm, the errors of *A* and *V* are below 2% and 9%, respectively, compared with using a standard micropipette aspiration method. We also calculated the length of the steadily deformed cell (*L*), and the motion velocity of the cell (${u}_{c}$) using the sequential images of the steadily deformed cell moving through microtubes. Together with the diameter of the tube (*D*), and the fluid velocity inside the channel under the same negative pressure but without cells (${u}_{0}$), we used a machine learning method to predict the membrane elastic shear modulus of RBCs (${E}_{s}$). In this study, we utilized a neural network method trained with a backpropagation (BP) algorithm, where a three-layer BP network was adopted to extract the shear modulus from the geometric and dynamic parameters observed in the experiments.

### Experimental setup

In the present study, sequential images of each deformed RBC during mechanical cycling are acquired under bright field microscopy using a 60 × objective (U Plan super apochromat; 60 × 1.35 NA numerical aperture) with a double mirror, and recorded using a CMOS camera (Phantom 410L, Vision Research), as shown in Fig. 1a,b. The camera runs at 200 frames per second (fps) with an exposure time of 1 ms and the imaging resolution of 250 nm, with the pixel size of 0.167 μm. A tubular channel with an inner diameter in the range of 3.0–3.2 µm was manufactured by a pipette puller (P-97, Sutter Instrument) and a microforge (Narishige, MF-830), and filled with a 1% BSA-PBS solution before use. A micromanipulator (Eppendorf, TransferMan 4r) was used to hold and move the channel precisely at the nanometer level. A chamber with a thickness of 2 mm and a centimeter-range length and width was prepared and placed on the stage of an inverted microscope (Olympus, IX73) as a reservoir of dilute RBCs suspensions. The upper and lower parts of the chamber were coverslips with 0.16 mm thickness, and cured silicone (PDMS, Polydimethylsiloxane) is used as a support structure on both sides of the coverslip at bottom. The chamber was filled with RBCs suspended in PBS and the volume fraction of RBCs was about 0.01%. Under the surface tension of the liquid, the liquid can be stably stored in the chamber. The chamber was closed above and below, and only small areas of the side was exposed to air. The semi-enclosed structure prevent evaporation and change of protein content.

The mechanical cycling of RBCs was conducted by first connecting the tubular channel to a negative pressure (P_n_ = − 847 Pa) after approaching a single RBC to it with micromanipulator. While one cell was aspirated into and flowed through the microtube, the negative pressure was held for 6 s, such that a steadily deformed profile of the RBC in the tube forms after the sudden contraction from the chamber to the microtube (Fig. 1b). Second, we switched the three-way valve to shift the hydraulic pressure in the microtube from the negative to a positive pressure (P_p_ = 480 Pa), and the pressure was held for 12 s, during which the steadily deformed RBC was returned into the chamber from the microtube after the sudden expansion (Fig. 1b). After being ejected from the microtube, RBCs take time to recover from a deformed shape to stress-free profile, while damping the energy of deformation through the viscous effect of the surrounding liquid (Fig. 1b). During one complete round of 18 s in the present mechanical cycling model, each RBC takes approximately 2 s in the entrance region of the tube for aspiration, ~ 4 s to steadily deform and move in the microtube, ~ 4 s to be ejected back into the chamber from the tube, and around 8 s to recover before the next round of cycling. In our experiments that the pressure gradient is about 1 Pa/μm and similar to the physiological condition of IES. The RBC velocity in microcapillary is about 500 μm/s and is similar to actual velocity in capillaries.

### Numerical method

Numerical simulations for single RBC squeezing into and exiting a microtube of 3 $\mathrm{\mu m}$ diameter illustrate the membrane stress distributions during mechanical cycling (Fig. 1c,d) using the immersed boundary methods as described in Wang et al.^25^ and Jing et al.^26^. Either passing through in a splenic slit in vivo or through a micro tube in vitro, RBCs’ membranes experience one round of stress variation from head to tail entirely as shown in the simulation results of Fig. 1d. Near the entrance of the slit or tube (where s = 0 in Fig. 1d) the gradient of stress distribution along membrane is often the largest, while the stress on the membrane at the head or tail of the deformed cell is the most concentrated as illustrated in Fig. 1c. Although RBCs’ membranes experience twice of the stress variations in one time of cycling using the present fatigue model while cells are aspirating into and pushing out the micro tubes comparing with that of a cell passing through IES unidirectionally, no additional or different stress on the cell membrane is created, because the distributions of membrane stress during aspirating or pushing cells are almost identical with negligible differences from the front to the rear ends as shown in the numerical simulations quantitatively. RBCs are drawn into the microtubule repeatedly in different directions randomly during their cycling, which means that any defects in the membrane can react to the stressing process randomly.

Compared with other microfluidic devices^20^ the major difference is that the stress distribution on the membrane of cell under test is axisymmetric using micro tubes. Researchers often ignore that using a conventional microfluidics with rectangular channel, the stress on cell membrane near the four corners of the cross section of the channel present significant stress concentrations, especially when a narrow slit is set for a cell to passing through which may introduces extra stress concentration near the corner of the rectangular channels. One major advantage of using the present setup with micro tubes instead of microfluidics with rectangular channels is that the pathway for the cell under test is simple at the entrance of the micro tube, so that the pressure gradient at this crucial opening can be accurately fixed to about 1 Pa/μm and similar to the physiological condition of IES, however, this physiological pressure gradient at the slits in a microfluidic channel is often difficult to fix because along the flow direction in the channel there are many inevitable fluctuated pressure losses from various connectors and complicated structures before a cell reaching the crucial slits. Another benefit using the micro tubes is that we could measure the surface area and volume of RBCs accurately during each cycle of fatigue based on images of the deformed cells under axisymmetric assumptions.

Limitations of the present simple in vitro model include that under the strong light for visualization under microscopy, mechanical properties of RBCs might change considerably after 2 h of experiments due to the thermal effects of the chamber and ATP depletion. It should be noted that the approximately 200 cycles in our model are not enough to fully replicate the entire process of RBC aging. Nonetheless, our model can evaluate the primary characteristics of changes in mechanical properties during the initial stages of RBC senescence.

### Image processing

To measure cell parameters, the background subtraction method was used to identify RBC contours. An image of the microtube without moving cells was selected as the background image. Threshold processing was used to remove the noise that remained in the stationary background region, which was irrelevant for detecting the moving targets. The length of the steadily deformed cell (*L*) was calculated by RBC contours and the velocity of the cell (${u}_{c}$) was calculated as the displacement of the cell centers over time.

We assumed that the deformed RBCs under the low Reynolds number flow in the steady-state region were axisymmetric. Accordingly, the surface area and volume of the RBCs were determined according to the cell contours; i.e. the surface area (*A*) and volume (*V*) were the summations of the surface areas and volumes of the elementary cones, respectively, as follows:

$$A=\sum_{i=1}^{n}{A}_{i}=\sum_{i=1}^{n}\frac{\pi }{2}\left({D}_{lefti}+{D}_{righti}\right)\sqrt{{\left(\frac{{D}_{lefti}}{2}-\frac{{D}_{righti}}{2}\right)}^{2}+{h}^{2}},$$

$$V=\sum_{i=1}^{n}{V}_{i}=\sum_{i=1}^{n}\frac{\pi h}{12}\left({{D}_{lefti}}^{2}+{D}_{lefti}{D}_{righti}+{{D}_{righti}}^{2}\right),$$

where ${D}_{left}$ and ${D}_{right}$ are the diameters of frustums on the left and right sides, respectively, and $h$ is the thickness of the discretized truncated cone.

### Sample preparation

Blood from healthy adult volunteers with an average age of 27 (in the range of 23–31) was collected using a finger prick with informed consent. The blood was diluted 1:2000 (approximately 2 million RBCs per milliliter) in phosphate saline buffer (PBS; CaCl2-free, MgCl2-free; pH 7.4; Gibco™) containing 1% (w/v) bovine serum albumin (BSA; Sigma–Aldrich) to prevent adhesion to other cells and device walls. All blood samples were stored at 4 °C before the experiment and tested within 12 h of the sample being drawn from the finger.

All methods were performed in accordance with the relevant guidelines and regulations and informed consent was obtained from all participants. All experiments were approved in advance by the Ethics Committee of Science and Technology of Shanghai Jiao Tong University.

### Statistical study

RBCs were individually tracked as a function of the loading cycle number during the course of the experiments. Statistical analyses were performed using SPSS software (IBM SPSS Statistics 22, USA); p values and the correlation coefficient r were calculated by two-tailed Pearson correlation tests between different physical parameters of the same cell and loading cycles. Univariable linear correlation analyses were performed to test the univariate associations of physical parameters and loading cycles, and p values of less than 0.05 were considered to be statistically significant.

## Results

### Shape transitions of RBCs during mechanical cycling

**Figure 2:**
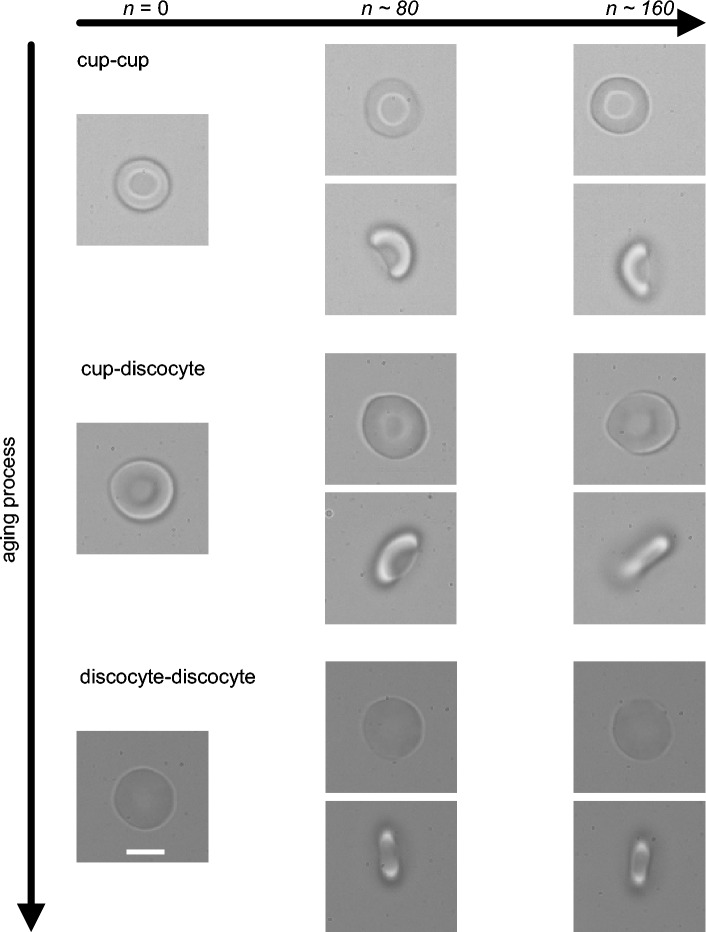
Typical shape transitions of RBCs during mechanical cycling. Images illustrate the shape transformation of a single RBC with loading cycles (n = 0, n $\approx$ 80, n $\approx$ 160). In the C–C sequence, the shape maintains a cup shape (upper, plan view; lower, side view). In the C–D sequence, the shape maintains a cup style at the cycle number n = 0 and around 80 (n $\approx$ 80), but transfers to biconcave when n approaches cycle n $\approx$ 160 (upper, plan view; lower, side view). In the D–D sequence, the shape maintains a biconcave shape (upper, plan view; lower, side view). Scale bar, 5 µm.

In the present experimental data obtained, RBCs were from the fingertip blood of several healthy adult donners. Under the microscope, we found that discoid biconcave-shaped RBCs constituted the largest portion, and cup-shaped cells made up almost all of the remaining portion, while rare-shaped RBCs such as acanthocytes are barely present. The average percentages of the RBCs in biconcave and cup shapes are 68.3% and 31.7% respectively. As the number of cup-shaped cells is much larger than the number of reticulocytes from a healthy donor, which is typically in the range of 0.2-2% of all RBCs in a normal state, we regard the cup-shaped RBCs mainly as matured erythrocytes with a little bit larger surface area to volume ratio than discoid biconcave-shaped RBCs. The present mechanical cycling model simulates mechanical fatigue, and enables the observation of transitions of stable shapes between the cup-shaped and biconcave-shaped RBCs in vitro (Fig. 2). Based on the initial shapes and the stable morphologies achieved after a couple of hundred rounds of mechanical cycling, shape transformations of RBCs are sorted into three categories. These are the transformation from cup-shaped to cup-shaped (noted as C–C) in which RBCs maintain their cup-shape during mechanical cycling, but the concavity height decreases gradually with the decrease in surface area; the transformation from cup-shaped to biconcave discocyte (C–D) in which the profiles of RBCs transfer from the cup-shaped initially to biconcave-shaped, and the transformation from discocyte to discocyte (D–D) in which RBCs retain the discoid biconcave shape but the cell sphericity steadily increases with a decrease in the cell surface area.

Since cup-shaped cells on average have a larger surface to volume ratio than discoid biconcave-shaped ones, and the surface area of RBCs often decreases monotonically during RBC aging^2^, the present experimental data from our in vitro mechanical fatigue tests suggests that mechanical fatigue induces shape transitions unidirectionally from cup-shaped to biconcave-shaped during the aging process. RBCs in the D–D group have the largest portion among all RBCs sampled from healthy individuals.

### Variations of RBC mechanical properties during mechanical cycling

**Figure 3:**
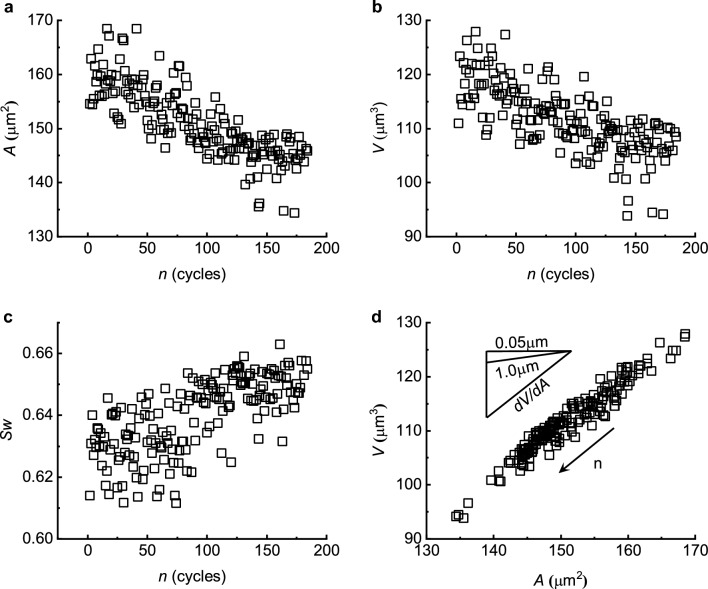
Typical variations of mechanical properties of different RBCs during mechanical cycling. Changes in surface area (**a**), cell volume (**b**), the swelling ratio (**c**) and ratio of volume to surface area with cycles (**d**). Oblique lines in the triangle in (**d**) represent the ratio of volume to surface area, where volume losses with 50 or 1000 nm spherical vesicles are considered (upper line: 0.05 μm; middle line: 1 μm; bottom line: the present experimental results during cycling).

Mechanical properties of each RBC including the surface area (*A*), volume (*V*), and membrane shear modulus (${E}_{s}$) were deduced and recorded for each cycle during mechanical cycling. As shown in Fig. 3, the cell surface (Fig. 3a) and volume (Fig. 3b) decrease linearly with the cycling time. Using the swelling ratio defined as $\mathrm{Sw}=3\mathrm{V}/4\pi {R}^{3}$, where $\mathrm{R}=\sqrt{A/4\pi }$ for cell sphericity (i.e. $\mathrm{Sw}=1.0$ for a sphere and $\mathrm{Sw}=0.64$ for a typical healthy RBC with discoid biconcave profile), Fig. 3c indicates that RBCs tend to round up with the reduction in surface area during mechanical cycling, while the change in the cell volume to surface area (dV/dA) is maintained constant (Fig. 3d). Vesiculation is the main manifestation of membrane loss during cell aging^8,27^. The shed vesicles typically have a size of 50-100 nm and appear as spherical particles due to the surface tension effect. The inset of Fig. 3d shows references for the change of volume to the loss of surface area that if RBCs were losing cell volume only by the spherical vesicles with the diameter of 0.05 μm or 1.0 μm during cycling, the relationship of volume to the surface area after shedding vesicles should be parallel to the lines marked with 0.05 μm or 1.0 μm respectively. Compared to the slope of the volume to surface area in Fig. 3d, the loss of volume of a RBC during mechanical cycling is significantly larger than that encapsulated by the shed vesicles, which indicates that with the membrane loss during mechanical cycling, RBCs modulate their morphologies in order to maintain the biconcave shape by losing more cytoplasm and leaving a larger redundant surface area to maintain their superb deformability.

**Figure 4:**
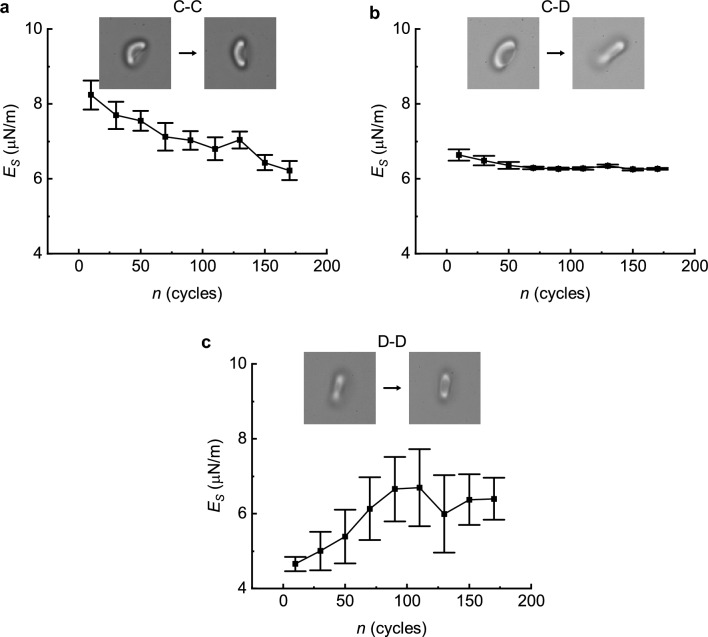
Changes in the membrane shear modulus during mechanical cycling in different groups of shape transformations (mean ± standard error), where (**a**) C–C denotes RBCs remaining in cup shape, (**b**) C–D for the transformation from cup to discocyte shapes and (**c**) D–D for RBCs remaining in discocyte shapes. Insets illustrate the typical morphological changes of different groups during cycling.

Although the surface area and volume reduce monotonously during mechanical cycling, the variations of the membrane shear modulus present a dependence distinctly on the shape transformation of RBCs. Using the fatigue model in vitro with microtubes, the aging processes of these RBCs of cup-shaped and biconcave-shaped are furtherly categorized into three different groups. We found that although the stiffening of cells during fatigue is monotonous for biconcave-shaped cells, but the cup-shaped cells could be either softening or stabilizing depending on their initial as well as final shape profiles after around 200 times of stressing-relaxation cycles through narrow micro tubes. As shown in Fig. 4, the shear modulus of those RBCs decreases evidently when retaining their cup shape, marked as C–C, during mechanical cycling (Fig. 4a). Shear modulus of those RBCs decreases slightly, but almost maintains constant while they transform from cup shape to discocyte marked as C-D (Fig. 4b); the shear modulus increases with a large scatter during mechanical cycling for those RBCs maintaining biconcave shape during cycling (Fig. 4c). To the best of our knowledge, this is the first result to reveal, with the aid of a mechanical cycling model, that the stiffness of RBCs varies across different phases of mechanical fatigue induced cell aging from cup-shaped to biconcave-shaped.

**Figure 5:**
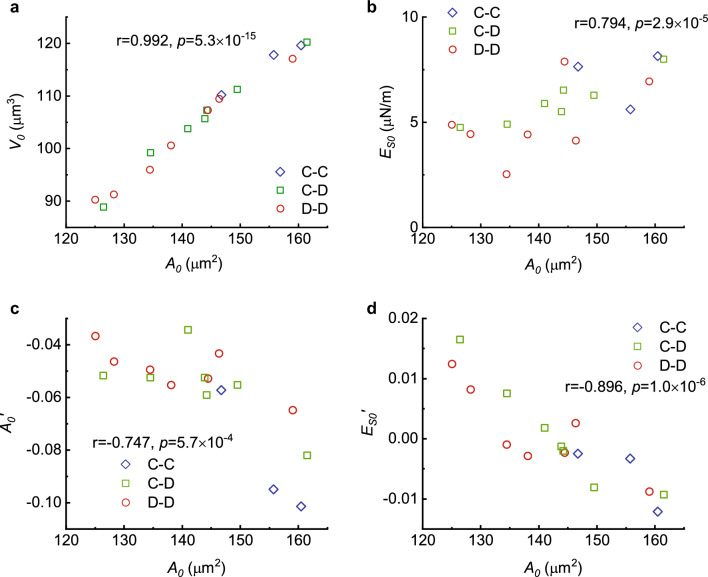
Correlations of the mechanical properties of RBCs to their initial surface areas. (**a**) Distribution of initial volume ${V}_{0}$ to initial surface area ${A}_{0}$ before mechanical cycling. (**b**) Distribution of initial membrane shear modulus ${E}_{S0}$ to initial area ${A}_{0}$. (**c**) Change rates of surface area ${{A}_{0}}^{\mathrm{^{\prime}}}=dA/dn$ during cycling to their initial surface area ${A}_{0}$. (**d**) Change rates of the shear modulus ${E}_{s0}^{^{\prime}}=d{E}_{s}/dn$ during cycling with respect to initial surface area ${A}_{0}$. Here,* r* and *p* in the figures denote the correlation coefficients and their significance between the variables chosen as coordinates, respectively. Blue, C–C; green, C–D; red, D–D.

Further detailed correlation analyses on the mechanical properties of RBCs are illustrated in Fig. 5 that cell volumes are closely related to their surface areas not only initially before mechanical cycling (Fig. 5a) but also at any given time during cycling (Fig. 3d). Among all mechanical properties measured in the present work, the surface area of RBCs, *A,* is selected as a key indicator for cell aging during mechanical cycling, not only because the surface area decreases monotonically with cell aging^2^ but also because it is more independent than the volume of cells with respect to their mechanical or chemical environments at the cellular level^6,10^. Among the time series of surface area ${A}_{n}$ acquired during the present experiments of mechanical cycling, where the subscript *n* stands for the cycling times, the initial surface area ${A}_{0}$ of a RBC before cycling, $n=0$, is an important parameter that in an average sense indicates the current age of the RBC before mechanical cycling. Our experiments demonstrate that besides the initial cell volumes (Fig. 5a) and membrane shear moduli (Fig. 5b), the change rates of the surface area ${{A}^{^{\prime}}}_{0}$ and shear modulus ${{E}^{^{\prime}}}_{S0}$ are all strongly correlated with their initial surface areas ${A}_{0}$ (Fig. 5c,d) as well.

We only acquired data from 17 cells in the present work, among which 10 of them were initially cup-shaped (3 of them kept cup shape, 7 of them shifted from cup-shaped to biconcave-shaped), and 7 of them kept biconcave-shaped throughout the mechanical cycling. The average and standard deviation of volume, surface area, initial shear modulus is 105.7 $\pm$ 9.8, 143.5 $\pm$ 11.2, 5.8 $\pm$ 1.5 respectively. Since the present work is a single-cell based longitudinal study versus RBC mechanical fatigue cycles, 17 is a relatively small number of the total samples but as shown in Figs. 4 and 5, the standard deviations of the measured variables are quite small which suggests that our observed trends are reliable.

### Mathematical models for surface area and shear modulus evolution of a single RBC during mechanical fatigue

Benefiting from the accurate mechanical properties of RBCs measured during each cycling with the present mechanical fatigue model in vitro, we established a mathematical model for the evolution of surface area and shear modulus of single RBCs under mechanical fatigue.  In our experiments, each RBC underwent approximately 200 cycles of mechanical fatigue within 2 hours at room temperature. On average, the surface area of RBCs decreased by 10% during this fatigue process, as illustrated in Fig. 3a. The present experimental data supports that the surface area of RBCs loses exponentially during mechanical fatigue based on the following two related estimations. Firstly, a healthy RBC in the human body passes through splenic inter-endothelial slits (IES) around 1500 times in total approximately during its lifespan about 120 d on average, because although the lung-to-lung circulation period of blood in healthy subjects is about 55 s, but during one round of peripheral blood circulation only about 5% of arterial flow enters the spleen through the splenic artery, and among which only 15% engages in the open and slow circulation in the red pulp and crosses the unique structure of the IES according to Henry et al.^28^. Secondly, as RBCs age, they tend to lose approximately 20% of their surface area. This estimation came from Waugh et al.^2^, who found that by separating young and aged RBCs based on their mean cellular hemoglobin concentration (MCHC), the surface area of young RBCs (with MCHC around 31 g/dL) was approximately 18% larger than that of aged RBCs (with MCHC > 37 g/dL). It’s worth noting that “young” and “aged” here refer to different stages of RBCs during their roughly 120-day lifespan. That is, at the beginning of 200 rounds of mechanical cycling as performed in the present experiments, freshly-drawn RBCs on average have already lost half of their spendable surface areas during the whole aging process that roughly requires 1500 rounds of cycling.

Assuming that the reduction of surface area $A$ obeys an exponential decay during mechanical cycling, the surface area at any given round of mechanical cycling *n* is formulated as

1 $${A}_{n}=a{e}^{-k(n+{n}_{0})}+{A}_{\infty },$$

where ${A}_{n}$ is the surface area of a RBC after the $n$ th cycling. ${A}_{\infty }$ is the limit remaining area of an RBC before it is eliminated from the circulatory system at the end of its lifespan, which is the final surface area that an RBC gradually decays to after an infinite number of cycles theoretically. Regarding the times of cycling $n$ as a temporal index, the surface area initially before cycling is given by ${A}_{0}=a{e}^{-k{n}_{0}}+{A}_{\infty }$ which presents the current status of the cell. When $n=-{n}_{0}$, meaning if we could trace the surface area of an RBC from the current status ${A}_{0}$ back to when the cell was born, the surface area of the newly born cell should be ${A}_{b}=a+{A}_{\infty }$. Then, the biophysical meaning of $a$ is clear that it stands for the total surface area that a single RBC can lose throughout its whole life cycle from birth to death, and $a{e}^{-k{n}_{0}}$ stands for the remaining surface area that an average RBC is going to lose since its current state. $k$ is the coefficient for the decay of surface area, and we assume that $k$ is identical for all RBCs from the same individual. Among multiple parameters $\left({A}_{n},n,a,k, {n}_{0},{A}_{\infty }\right)$ in the mathematical model for a single RBC, ${A}_{n}$ and $n$ are measured directly from the present experiments, and $k$ and ${A}_{\infty }$ are obtained by data analysis, whereas $a$ and ${n}_{0}$ cannot be decided explicitly. Here, n is supposed to be a continuous parameter, but it is discretized in the experiments by each round of cycling and measurement.

Multiple parameters $\left(a,k, {n}_{0},{A}_{\infty }\right)$ with deterministic biophysical meanings are utilized in the present mathematical model, but the parameter matrix is difficult to solve explicitly for any single RBC with the present experimental data due to the heterogeneity of the cells. Using the characteristics of the exponential function, the change rate of cell surface is given as follows

2 $${{A}^{^{\prime}}}_{n}=-ka{e}^{-k\left(n+{n}_{0}\right)}=-k\left({A}_{n}-{A}_{\infty }\right), \mathrm{and\, }k=-\frac{{{A}^{^{\prime}}}_{n}}{\left({A}_{n}-{A}_{\infty }\right)}.$$

Theoretically, a set of equations for ${{A}^{^{\prime}}}_{n}$, ${A}_{n}, {A}_{\infty }$ can be formulated with the experimental data during cycling. However, the fluctuations of ${{A}^{\mathrm{^{\prime}}}}_{n}$ and ${A}_{n}$ observed in the experimental data during successive rounds of cycling (Fig. 3a) affect the accuracy of the evaluation, which come from both the instability of fluid flows in the micro channel with a deforming cell and errors in the image processing. Here, to take advantage of the longitudinal experimental data of a single cell during cycling, but avoid local fluctuations, we performed the following treatments.

Firstly, we calculate the change rate of the surface area of a single RBC by fitting all experimental data points along the 200 cycles. Then use the resulting average value, denoted by $<{{A}^{\mathrm{^{\prime}}}}_{n}>$, as the initial change rate, ${{A}^{\mathrm{^{\prime}}}}_{0}\approx <{{A}^{\mathrm{^{\prime}}}}_{n}>$.

**Figure 6:**
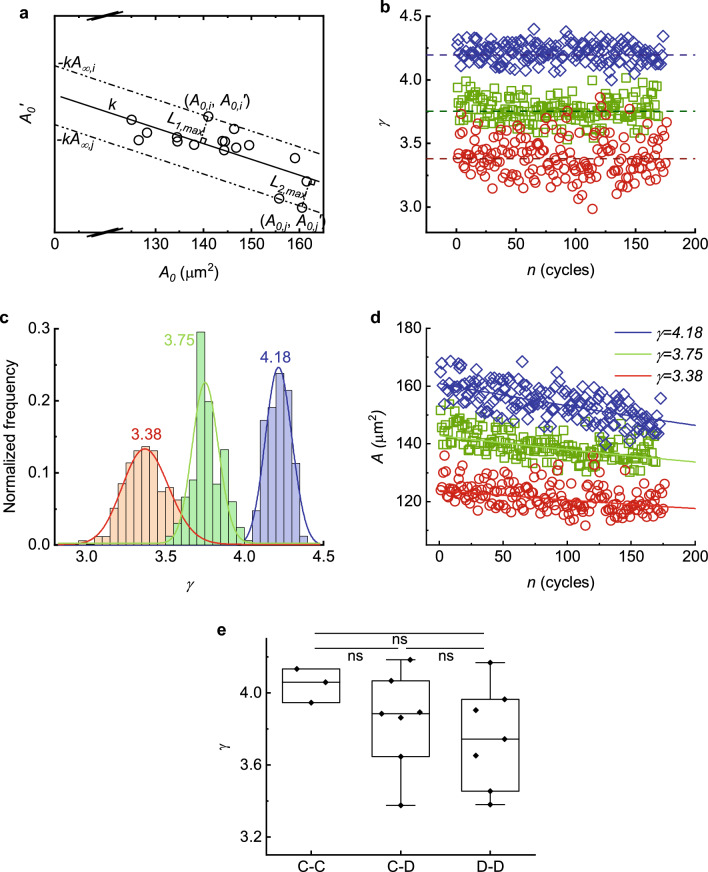
Mathematical modeling of single RBC area dynamics. (**a**) Least squares fitting of all RBCs data from single individual in plane of (${{A}^{^{\prime}}}_{0}, {A}_{0})$ obtain $k$ and ${A}_{\infty }$. (**b**) $\gamma$ variation as a function of the number of cycles. (**c**) Distributions of $\gamma$ for the same RBC during the cycling test. (**d**) Model fits to area reduction as a function of the number of cycles and comparisons of cell area between different values of $\gamma$. Scattered points represent the experimental data (blue: $\gamma$ = 4.18; green: $\gamma$= 3.75; red: $\gamma$= 3.38 in (**b–d**). (**e**) Comparison of the $\gamma$ for three distinct groups of cells. ns means no significance in statistics.

Although we assume that the surface area decays exponentially over the whole aging process, in the present experiments in vitro, each single RBC experiences only about the one-tenth of its entire life cycle. As a result, the rate of change could be approximated under a linear assumption, with acceptable deviations within the range. Secondly, we assume that the value of *k* must remain constant for all RBCs from a particular individual. This ensures that the final surface areas of the RBCs from the individual, denoted as ${A}_{\infty }$, can be calculated using Eq. (2), which states that ${A}_{\infty }=({{A}^{^{\prime}}}_{0}+k{A}_{0})/k$. That is, $k$ is fitted using the least squares method with all data of RBCs from a single individual in the plane of (${{A}^{^{\prime}}}_{0}, {A}_{0})$, as shown in Fig. 6a. In this manner, the $k$ for a group of samples from each particular individual and the ${A}_{\infty ,i}$ for each individual RBCs among these samples can be characterized.

To describe the current health status of a single RBC quantitatively, an ensemble parameter $\gamma$ is defined in Eq. (3) by taking the natural logarithm of both sides of Eq. (1).

3 $$\gamma =\mathit{ln}a-k{n}_{0}=ln\left({A}_{n}-{A}_{\infty }\right)+kn.$$

This parameter involves the potential surface area to be lost, $a$, the coefficient for exponential decay of surface area, $k$, and its current cycling index, ${n}_{0}$. Using the experimental data of an RBC at each fatigue cycle, $\gamma$ of a given cell is estimated with $\gamma =ln\left({A}_{n}-{A}_{\infty }\right)+kn$ as shown Fig. 6b. It is observed that $\gamma$ fluctuates with the fluctuations of measured ${A}_{n}$.

The available experimental data provide evidence to support the utilization of $\gamma$ as an ensemble parameter for a single RBC. The medians of $\gamma$ in each cell during mechanical cycling appear to be largely independent of the number of cycles, as illustrated in Fig. 6b. Additionally, the transient values of $\gamma$, which are calculated using the instant experimental data at each cycle, conform to a Gaussian distribution, as depicted in Fig. 6c. Using the medians of $\gamma$, the decay of the surface area of each RBCs from the same individual during mechanical fatigue can be clearly categorized (Fig. 6d). After comparing the $\gamma$ values for three different groups of shape transformations, statistical analysis showed no significantdifferences (Fig. 6e). Considering the measurement errors in present experiments, our results indicate that the ensemble parameter $\gamma$ of a single RBC, as determined by our mathematical model, is capable of providing a quantitative distinction between the mechanical cycling behaviors of each cell.

The change of membrane shear modulus of a single RBC during the mechanical fatigue process is more complicated than the monotonical decay of the surface area, which varies in three distinct manners associating with the three different cell shape transformation as shown in Fig. 4. And Fig. 5d presents that the change rate of the shear modulus of a single RBC at the beginning of mechanical cycling, ${{E}^{^{\prime}}}_{0}$ is linearly correlated with its initial area, ${A}_{0}$, as

4 $${E{s}^{^{\prime}}}_{0}=g{A}_{0}+C,$$

where $g$ is the linear change rate, and $C$ is a constant. We generalize this linear correlation between the change rate of the membrane shear modulus and the surface area from the initial state of cells to any current states of RBCs, and assume that $g$ stays constant for all RBCs from one individual as a personal characteristic as

5 $${E{s}^{^{\prime}}}_{n}=g{A}_{n}+C.$$

Taking Eq. (1) into (5), and integrating the equation with the initial conditions ${Es}_{n}={Es}_{0}$ and ${A}_{n}={A}_{0}$ at $n=0$, the membrane shear modulus along the mechanical fatigue process is formulated as

6 $${Es}_{n}=-\frac{ag}{k}{e}^{-k\left(n+{n}_{0}\right)}+\left(g{A}_{\infty }+C\right)n+{Es}_{0}+\frac{ag}{k}{e}^{-k{n}_{0}},$$

where $C$ is a constant that assumes different values for different cells. This formulation of the membrane shear modulus consist of an exponential decay part and a linear growth part together, which jointly determine the variation of the shear modulus with respect to mechanical fatigue at any given cycling time $n$.

The model presented in Eq. (6) illustrates three distinct tendencies for the change of the shear modulus, as demonstrated in Fig. 5. These tendencies are dependent on two factors: ${A}_{0}$ and *g*. If ${A}_{0}<-C/g$, the shear modulus initially decreases with mechanical cycling (${{Es}^{^{\prime}}}_{0}<0$). Conversely, if ${A}_{0}>-C/g$, the shear modulus increases (${{Es}^{^{\prime}}}_{0}>0$). If ${A}_{0}=-C/g$, the shear modulus remains constant ($Es_{0}^{^{\prime}} = 0$). By expressing the shear modulus in terms of the surface area,

7 $${Es}_{n}=-\frac{g}{k}\left({A}_{n}-{A}_{\infty }\right)+\frac{1}{k}\left(g{A}_{\infty }+C\right)\left\{lna-k{n}_{0}-\mathrm{ln}\left({A}_{n}-{A}_{\infty }\right)\right\}+{Es}_{0}+\frac{ag}{k}{e}^{-k{n}_{0}},$$

which contains the changes in the shear modulus with shape transformations during the mechanical fatigue processes of a single RBC.

**Figure 7:**
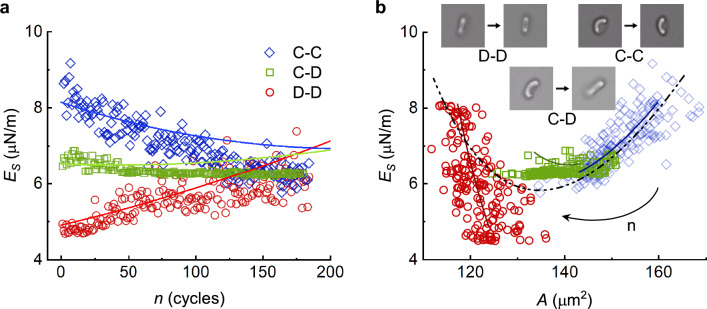
Comparison of mathematical model (solid line) of the membrane shear modulus with experimental data (scattered points) under different shape transformations. (**a**) Shear modulus variation during mechanical cycling. Different colors and line styles represent different shape transformations associated with the cycling processes. (**b**) Relation of shear modulus to surface area during mechanical cycling with three different cells under different shape transformations during cycling from cup-shaped to cup-shaped (diamands), cup-shaped to discoid biconcave-shaped (squares), and discoid biconcave-shaped to discoid biconcave-shaped (circles) respectively. The arrow illustrates the direction of the aging process using the number of cycling *n* as an index, the dash-dot-dot line presents an expectation for one cell during a long-cycling process based on Eq. (7).

Figure 7a shows that Eq. (6) approximates the experimental data closely with the $g$ determined by adopting the change rates of the shear modulus of multiple RBCs as a constant from the same individual. Figure 7a also reveals that during the fatigue process of a single RBC, the membrane shear modulus first decreases while its shape profile transforms from cup-shaped to discocyte-shaped, and then increases as it maintains discoid biconcave shapes.

As shown in Fig. 7b, the solid lines and scatter points present the model prediction and experimental data of three different cells under three distinct shape transformations respectively, which illustrates that Eq. (7) is able to predict the change of membrane shear modulus associate with the variation of surface area during the fatigue process approximately. Figure 7b supports that a single RBC often contains larger surface area with a cup shape, and starts to lose the surface area with a fading of the cup-shape, meanwhile, the membrane shear modulus decreases. The softening of the RBC membrane ceases as cup-shaped cells transform into a discoid shape. During this transformation, RBCs continue to lose surface area, but the shear modulus remains relatively constant and does not exhibit significant changes around its minimum value throughout its lifespan. After RBCs obtain their discoid biconcave shape, RBCs start to stiffen during the mechanical fatigue process such that their surface areas reduce, and the membrane shear moduli increase.

In the present work, for each type of shape transformations including cup-cup, cup-discocyte and discocyte-discocyte, we conducted multiple cell experiments as shown in Fig. 4 and the overall trends of each type are consistent. Limited by the present experimental conditions, long term fatigue experiments for a single cell are not supported by the current setup, so that we could not observe a direct measurement of the whole fatigue process on a given cell. However, while we put three distinct categories together, it is clear that a parabolic shape is formed by the three groups of data jointly as shown in Fig. 7b. The physical models presented using Eqs. (1), (6) and (7) based on the present experimental data are simple. The parameters adopted here are biophysically meaningful which reflect certain basic characteristics of RBCs with individual differences of the cells and the fitness of each cell if it belongs to the same person. Although we could not present a whole aging process with a given cell currently, Fig. 7b and the dash-dot-dot line as the expected trend in the figure call for more advanced setups for more sophisticated single cell mechanical fatigue experiments.

## Discussion

Mechanical stimulation of RBCs is crucial for the aging of RBCs^29^. However, quantitative means to study these effects of mechanical stimulation on the mechanical properties of individual erythrocytes are still lacking. The fatigue model with microtubes proposed in this study not only rebuilds the circumferentially more symmetric stress stimulations while RBCs repeatedly pass through narrow capillaries and slits in vivo, but also characterizes the changes of mechanical properties of RBCs quantitatively after each fatigue event via hydrodynamic methods in situ.

During cycling, mechanical stimulation induces changes in RBC morphology, leading to a decrease in the area and volume of RBCs, as well as modifications in the shear modulus of RBC membrane. The alterations in the mechanical properties of RBCs observed in our study are similar to those observed in the aging process^11–14^. It is proposed that mechanical fatigue is the primary cause of the changes in mechanical degradation of RBCs observed in different age groups. Consequently, our model provides an explanation for how mechanical stimuli contribute to the aging process of RBCs. The present mechanical cycling model supports in-depth studies of the physiological and clinical issues related to the aging of RBCs.

The transformation of reticulocytes into matured erythrocytes undergoes three distinct phases, including R1 reticular structure (reticulate, containing RNA, rough surface), R2 cup-shaped structure (cup-shaped, containing RNA, rough surface), and matured RBC (double concave dish shape)^7^. Inspired by the morphological analysis of human erythrocytes^30,31^, researchers speculated that there is an R3 transition phase (cup, RNA-free, smooth surface) between the R2 and matured erythrocytes^32^. After conducting our current experiments, we have discovered that cup-shaped RBCs can transform into a biconcave-shaped discocyte through mechanical cycling. This process causes the shear modulus to decrease until the RBC stiffness reaches its minimum value during its lifespan. The discoid biconcave shape is one of the most stable shapes with the least amount of energy required during the shape transformation process of RBCs. Additionally, the RBC's deformability and stability increase during the transformation from cup to discocyte. These characteristics may help explain the observations on the maturation of reticulocytes, which show a rapid decrease in the area and volume of RBCs, while their cell deformability and mechanical stability increase^33^. Therefore, the experimental results along with the fatigue model suggest that the shape transformation from cups into discocytes is likely an intermediate phase in the maturation of reticulocytes into matured RBC, which is helpful to the study of in vitro–generated red blood cells.

RBCs participate in the pathological processes of numerous circulatory and metabolic diseases^34,35^. Combined with the mechanical stimulation, inflammation and other biochemical stimulations likewise affect the aging of RBCs, which provides great potential for disease diagnosis and treatment through monitoring of the mechanical properties of RBCs. Herein, we set up a mathematical model for the evolution of the surface area and shear modulus of a single RBC based on the aging process of RBCs in vitro. The parameters, including the surface area decay rate and the concomitant change rate of the shear modulus, are the intrinsic properties of RBCs which are potential indicators for describing the differences of RBCs in different diseases quantitatively.

For example, the decrease in deformability and membrane stability due to genetic factors in hemolytic anemias (e.g. thalassemia, hereditary spherocytosis) is often deteriorated by mechanical stimulations in the spleen^5^. Employing the proposed mathematical model, we may be able to quantitatively differentiate the intrinsic properties of RBCs in different hereditary blood diseases. Here, k describes the capability of RBCs resisting the surface area change for maintaining a steady-state, and g reflects the rate of the stiffening of RBCs due to cytoskeleton remodeling. These parameters suggest a novel approach to diagnose and monitor hemolytic diseases with abnormal variations of surface area and stiffness during mechanical cycling.

Another potential application is to check the effect of blood glucose on the aging of RBCs. Glycated hemoglobin (HbA1c) in diabetic patients is closely related to the RBC age^36^. With the ensemble parameter $\upgamma$ to estimate the ages of RBCs, HbA1c might be monitored more dynamically within a few hours using the present fatigue mode to accelerate the aging process under similar biochemical environments. However, all these potential applications require more data accumulation and more sophisticated experimental setups for long-term testing of single RBCs with appropriate control studies.

## Conclusions

We established a single RBC mechanical cycling model to mimic mechanical stimulation of RBCs in vivo. The model employs isotropic stress conditions for RBCs repeatedly passing through narrow lumens, such as small capillaries in microcirculation or IES in spleen, to effectively mimic the aging of cells through surface area loss. It furthermore provides in situ and precise measurements of the surface area and membrane shear modulus during each round of mechanical cycling. By utilizing an in vitro fatigue model, we have categorized matured RBC cycling processes into three distinct groups, based on their respective shape changes between cup-shaped and biconcave-shaped. Although the stiffening of cells during fatigue is monotonous for biconcave-shaped RBCs, the cup-shaped cells could be either softening or stabilizing depending on their initial as well as final shape profiles under the mechanical cycling. We also proposed mathematical formulations based on the experimental data to approximate and interpret the evolution of the mechanical properties of matured RBCs. Mathematical models for the change of surface area and membrane shear modulus of individual RBCs supports an ensemble parameter for estimating the health status of RBCs quantitatively.

## Data Availability

All data are available in the main text.
